# Regulation of transcriptome, translation, and proteome in response to environmental stress in fission yeast

**DOI:** 10.1186/gb-2012-13-4-r25

**Published:** 2012-04-18

**Authors:** Daniel H Lackner, Michael W Schmidt, Shuangding Wu, Dieter A Wolf, Jürg Bähler

**Affiliations:** 1 Department of Genetics, Evolution and Environment and UCL Cancer Institute, University College London, Darwin Building, Gower Street, London WC1E 6BT, UK; 2 Signal Transduction Program, Sanford-Burnham Medical Research, 10901 North Torrey Pines Road, La Jolla, CA 92037, USA; 3 The Salk Institute for Biological Studies, 10010 North Torrey Pines Road, La Jolla, CA 92037-1099, USA

## Abstract

**Background:**

Gene expression is controlled globally and at multiple levels in response to environmental stress, but the relationships among these dynamic regulatory changes are not clear. Here we analyzed global regulation during different stress conditions in fission yeast, ***Schizosaccharomyces pombe***, combining dynamic genome-wide data on mRNA, translation, and protein profiles.

**Results:**

We observed a strong overall concordance between changes in mRNAs and co-directional changes in translation, for both induced and repressed genes, in response to three conditions: oxidative stress, heat shock, and DNA damage. However, approximately 200 genes each under oxidative and heat stress conditions showed discordant regulation with respect to mRNA and translation profiles, with genes and patterns of regulation being stress-specific. For oxidative stress, we also measured dynamic profiles for 2,147 proteins, comprising 43% of the proteome. The mRNAs induced during oxidative stress strongly correlated with increased protein expression, while repressed mRNAs did not relate to the corresponding protein profiles. Overall changes in relative protein expression correlated better with changes in mRNA expression than with changes in translational efficiency.

**Conclusions:**

These data highlight a global coordination and fine-tuning of gene regulation during stress that mostly acts in the same direction at the levels of transcription and translation. In the oxidative stress condition analyzed, transcription dominates translation to control protein abundance. The concordant regulation of transcription and translation leads to the expected adjustment in protein expression only for up-regulated mRNAs. These patterns of control might reflect the need to balance protein production for stress survival given a limited translational capacity.

## Background

Cells adapt to stress or to changing environmental conditions by launching specialized gene expression programs that promote stress protection, homeostasis, and survival. Single-celled organisms like yeasts are particularly exposed to fluctuations in the environment that trigger a large common transcriptional response, called environmental stress response in budding yeast or core environmental stress response (CESR) in fission yeast [1,2]. The expression of hundreds of genes is either induced or repressed in response to different stress conditions in fission yeast [3-8]. Key regulators of this stress response programme are the mitogen-activated protein kinase (MAPK) Sty1/Spc1 and the b-ZIP transcription factor Atf1 [9,10].

Besides transcriptional regulation, it is clear that gene expression is also modulated at post-transcriptional levels in response to stress, including translational control [11-15]. Studies using translational profiling in budding yeast exposed to stress, such as changes in nutrients [16,17], treatment with rapamycin or heat shock [18], oxidative stress [19-21], or osmotic shock [22,23], have revealed global changes in translation and identified specific genes that are mainly regulated at the translational level. No comparable global analyses on translational control during stress have been reported in fission yeast.

Ultimately, proteins mediate the adaptation to stress, and it is expected that the levels of numerous proteins require rapid adjustment to different environmental conditions. It is not clear, however, how the changes in transcriptional and translational control are reflected in changes at the protein level. Poor correlations between mRNA and protein expression have often been reported [24-32], although some recent studies found much stronger relationships between the regulation of mRNAs and corresponding proteins [33,34].

Here we applied genome-wide translational profiling, combined with mRNA profiling, in fission yeast cells exposed to oxidative stress, heat shock, or DNA damage. Most stress-response genes were regulated in a concordant manner with respect to transcript levels and translational efficiency, which was evident for both stress-induced and -repressed genes. Several genes, however, bucked this trend and showed antagonistic regulation at the mRNA and translation levels. We also measured the dynamic response in the levels of more than 2,000 proteins during oxidative stress. A strong overall correlation was observed between transcriptional/translational induction of genes and induction of the corresponding proteins, but not between transcriptional/translational gene repression and protein profiles. Our data indicate that during oxidative stress in fission yeast, the changes in mRNA levels are the main determinant for changes in protein levels, while translational control plays a relatively minor role.

## Results and discussion

### Global translational control during environmental stress

To study translational changes in stress conditions, we prepared polysome profiles from unstressed cultures of fission yeast cells (control) and from the same cultures exposed for different times to oxidative stress, heat shock, and the DNA-damaging agent methylmethane sulfonate (MMS). The relatively mild doses of stress were chosen based on previous experiments to prevent substantial cell death [3,7]. Severe stress conditions lead to general translational repression [11-13,21,22,35]. We detected no substantial differences, however, between the overall polysome profiles from cells exposed to any of the stresses and unstressed control cells (data not shown), indicating that translation was not extensively altered on a global scale under the selected, relatively mild conditions [3,7].

**Figure 1:**
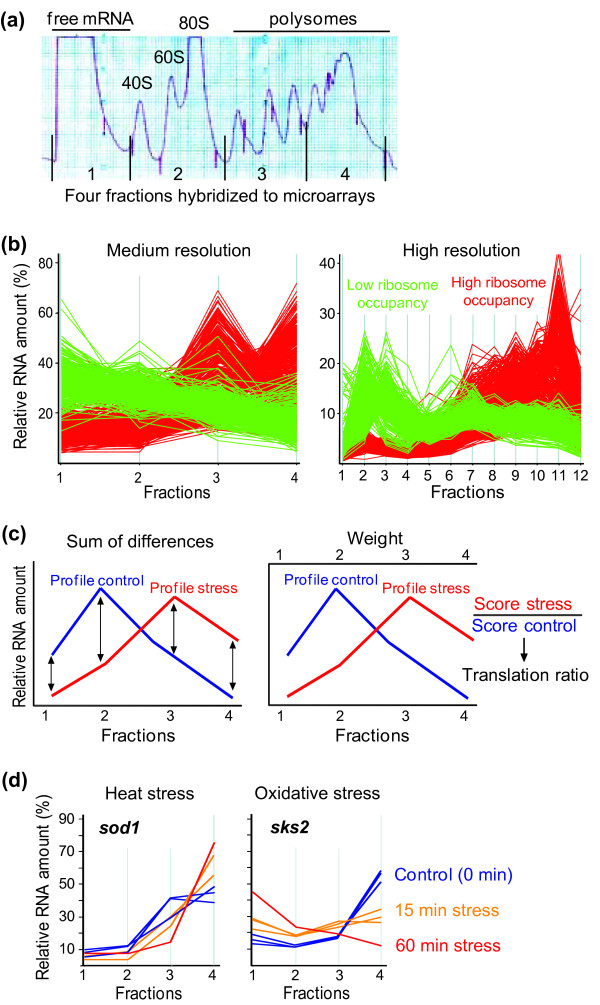
**Experimental layout and data analysis for translation profiling**. **(a) **Four equal mRNA fractions were each competitively hybridized to DNA microarrays against genomic DNA as common reference. Polysome profiles were prepared from unstressed control cells and from cells exposed to stress for 5, 15 and 60 minutes. **(b) **Comparison of polysome profiles for medium-resolution translation profiling with high-resolution profiling applied before [36]. The graphs show polysome profiles of the 10% mRNAs with the lowest ribosome occupancy (green) and the 10% mRNAs with the highest ribosome occupancy (red), measured by medium-resolution (left graph) and by high-resolution translation profiling (right graph). The profiles represent the average from three independent biological repeats. Ribosome occupancy was determined based on previous high-resolution translation profiling [36]. **(c) **Two complementary data analysis approaches to uncover translationally regulated mRNAs (Materials and methods). Left: the total difference for a given mRNA between the translation profile under stress and the translation profile in the control was calculated by summing up the differences of each fraction (indicated by arrows). Right: scores for each mRNA in each condition were calculated as described in Materials and methods. A translation ratio was then obtained by dividing the score of a given mRNA in a stress condition by the score of the same mRNA in the control condition (see Additional file 1 for data from translational profiling analysis). The combined lists from both approaches were then visually inspected to generate high-confidence, curated lists of translationally regulated genes (Table 1). **(d) **Left graph: translation profiles for ***sod1 ***mRNA before and after exposure to 39°C. Right graph: translation profiles for ***sks2 ***mRNA before and after exposure to H_2_O_2_. Blue lines, control (unstressed) samples; orange lines, 15 minutes after stress; red lines, 60 minutes after stress. Multiple lines of the same color represent translation profiles from independent biological repeats.

To analyze translational control, we extracted mRNA from four equal fractions throughout the polysome profiles, followed by labeling and hybridization onto DNA microarrays against labeled genomic DNA as reference (Figure 1a). To test whether translational profiles obtained from this medium-resolution approach reflected the data from high-resolution translation profiling using 12 fractions [36], profiles from the mRNAs with the highest and lowest ribosome occupancy were compared. There was good overall agreement between profiles from medium- and high-resolution translational profiling (Figure 1b): the mRNAs with the highest ribosome occupancy, corresponding to efficiently translated mRNAs, peaked in the higher fractions (red profiles in Figure 1b: fractions 3 to 4 in medium-resolution profiling; fractions 7 to 12 in high-resolution profiling), while the mRNAs with the lowest ribosome occupancy peaked at the lower fractions (green profiles in Figure 1b). Although somewhat less sensitive than high-resolution profiling, this comparison indicates that four fractions are sufficient to capture the essence of the translational activity, providing more information than simply comparing monosome with polysome fractions.

To identify mRNAs with altered translational profiles among the nuclear-encoded protein-coding genes, we initially applied a combination of two complementary automated approaches: 1) using a measure of the overall difference in mRNA profiles between stress and control samples; and 2) using a ratio of weighted translation scores between stress and control samples (Figure 1c; Additional file 1). These two methods gave largely (approximately 90%) overlapping yet complementary results, with the first one informing about overall differences and shifts in translational profiles and the second one informing about the levels and directions of translational changes. The profiles from these candidate mRNAs uncovered by either approach were then visually inspected to create a high-confidence set of translationally regulated mRNAs. Two typical examples of mRNAs showing translational control in response to stress are shown in Figure 1d: the *sod1 *mRNA (encoding a superoxide dismutase) is gradually shifted towards higher polysomal fractions in response to heat stress, reflecting translational up-regulation, while the *sks2 *mRNA (encoding a ribosome-associated molecular chaperone) is strongly shifted from the higher polysomal fractions towards fractions of free mRNA in response to oxidative stress, reflecting translational down-regulation. Table 1 shows the numbers of translationally regulated mRNAs in the different conditions, before and after filtering by visual inspection. Below, we will refer to the high-confidence data set as translationally regulated mRNAs.

**Table 1:** Numbers of mRNAs regulated after stress exposure

	Translation up	Translation down	mRNA up	mRNA down
H_2_O_2_, 15 min	26 (33)	75 (123)	171	38
H_2_O_2_, 60 min	191 (275)	566 (575)	455	374
39°C, 15 min	245 (280)	119 (140)	370	129
39°C, 60 min	99 (126)	146 (202)	291	334
MMS, 15 min	3 (19)	5 (27)	27	0
MMS, 60 min	27 (48)	60 (151)	147	15

Numbers of mRNAs among the 4,976 nuclear-encoded protein-coding genes that are regulated during the different stress conditions indicated at left. Translation up/down indicate the numbers of mRNAs that are up- or down-regulated, respectively, at the level of translation according to the criteria used (Figure 1c; Materials and methods), with numbers before filtering by visual inspection indicated in parentheses. mRNA up/down indicate numbers of mRNAs whose expression is up- or down-regulated, respectively, with respect to transcript abundance, based on a two-fold cutoff from mean expression ratio. Annotated lists of the translationally regulated genes are given in Additional file 2.

### Changes in mRNA levels and translation are globally coordinated

We detected 757 mRNAs that showed translational regulation during exposure to oxidative stress (at 60 minutes), and 364 mRNAs that showed such changes in heat stress (at 15 minutes), whereas only 87 mRNAs showed translational regulation after the exposure to DNA damage (Table 1; Additional file 2). Notably, the translationally up-regulated mRNAs for all three stress conditions were significantly enriched in up-regulated CESR genes (P ~ 9 × 10^-55 ^to 2 × 10^-22^), while translationally down-regulated mRNAs were significantly enriched in down-regulated CESR genes (P ~ 3 × 10^-173 ^to 5 × 10^-80^) [3,7]. These substantial overlaps between CESR genes and translationally regulated genes suggest that mRNAs regulated at the level of transcription are often also regulated in the same direction at the level of translation.

To directly compare the regulation of mRNA abundance with the regulation of translation, we also measured changes in relative mRNA levels by expression profiling of the same cell samples used for translational profiling. These mRNA expression data were highly similar to those previously described [3]. Table 1 shows the numbers of mRNAs whose levels substantially change in the various stress conditions, compared to the numbers of translationally regulated mRNAs. The response to heat was more rapid than the response to oxidative stress, which is reflected in both translational and mRNA up-regulation peaking at 15 minutes in the former and at 60 minutes in the latter stress. The response to DNA damage was relatively small compared to the other two stresses, both at the translation and mRNA levels. The down-regulation of translation was much more pronounced in oxidative stress than in the other stresses.

**Figure 2:**
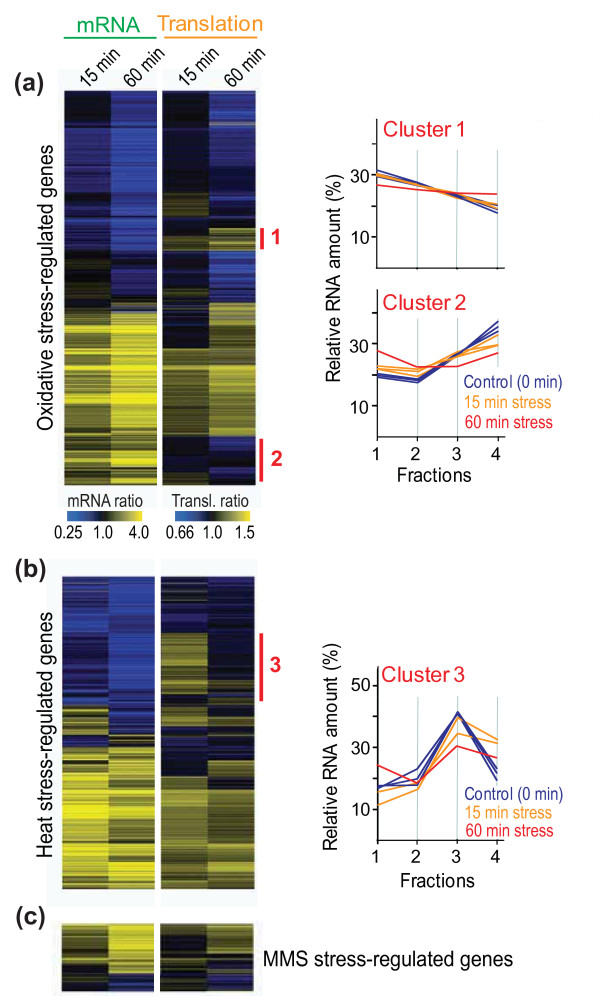
**Clustering of genes regulated during environmental stress**. **(a) **Hierarchical cluster analysis with columns representing experimental time points and rows representing the 1,355 genes that were regulated after exposure to H_2_O_2_, at the level of either mRNA and/or translation as defined in Table 1. Columns 1 and 2: mRNA expression levels after 15 and 60 minutes in stress, respectively, relative to expression in the same cells before stress are color coded as indicated at the bottom. Columns 3 and 4: translation ratios (Figure 1c; Additional file 1; Materials and methods) after 15 and 60 minutes in stress, respectively, are color coded as indicated at the bottom. Data from biologically repeated samples are averaged, with missing data in gray. The red bars indicate clusters 1 and 2 described in the text. The graphs at right show average translation profiles for the genes of clusters 1 and 2 before and after exposure to H_2_O_2_. Blue lines, control (unstressed) samples; orange lines, 15 minutes after stress; red lines, 60 minutes after stress. Multiple lines of the same color represent average translation profiles from independent biological repeats. Annotated gene lists of these clusters are provided in Additional file 3. **(b) **Cluster analysis as in (a) for the 1,071 genes that were regulated after exposure to 39°C, at the level of either mRNA and/or translation (Table 1). The red bar indicates cluster 3 described in the text. The graph at right shows the average translation profiles for cluster 3 genes before as well as 15 and 60 minutes after exposure to 39°C, with details as in (a). An annotated gene list of cluster 3 is provided in Additional file 3. **(c) **Cluster analysis as in (a) for the 233 genes that were regulated after exposure to DNA damage, at the level of either mRNA and/or translation (Table 1).

**Figure 3:**
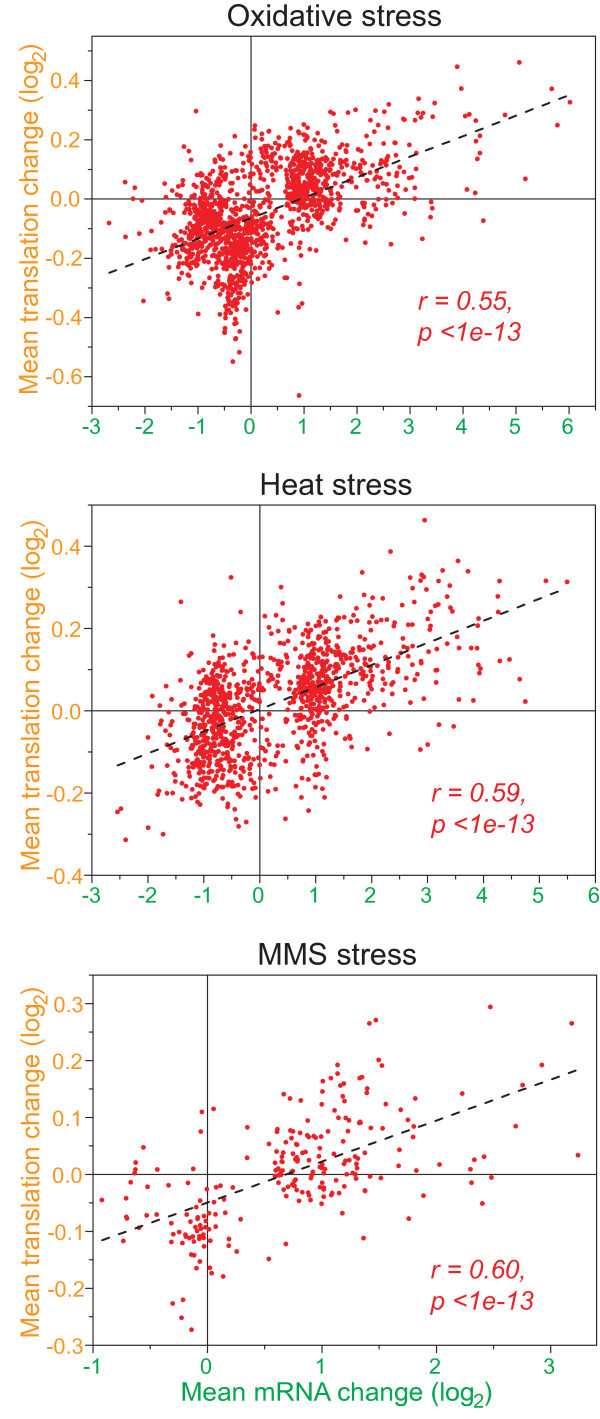
**Correlation between mRNA and translational regulation**. Scatter plots showing linear regressions of the average log_2 _changes in the oxidative (top), heat (middle), and MMS (bottom) stress time-course experiments. The gene lists were the same as those used for clustering in Figure 2 for the three stress experiments. The Pearson's correlations and probabilities (two-tailed test) are indicated for each experiment.

We then clustered the profiles of all the mRNAs that showed substantial changes in abundance and/or translation in the different stress conditions (Figure 2; Table 1). This analysis highlights the overall coordination between mRNA and translation profiles, which were typically regulated in the same direction. Accordingly, the average translation and expression ratios for the regulated mRNAs were significantly positively correlated in all stress conditions tested (Figure 3). Taken together, along with results on transcriptional control for these genes [3] (S Marguerat, K Lawler, A Brazma and JB, submitted), these data strongly support the idea that, during environmental stress in fission yeast, most mRNAs are regulated both at the level of transcription and at the level of translation in a concordant manner. Moreover, regulation at the level of mRNA turnover during oxidative stress is also globally coordinated with transcription (S Marguerat, K Lawler, A Brazma and JB, submitted). It is not known how such global coordination at multiple levels of gene expression is achieved by the cells.

### Genes differentially regulated at mRNA and translational levels

Although transcriptional and translational regulation were generally coordinated, we detected substantial gene groups that opposed this global trend (indicated as clusters 1 to 3 in Figure 2). The 74 genes of cluster 1 were translationally up-regulated in response to oxidative stress, most notably at 60 minutes, while their mRNA levels were down-regulated (Figure 2a; Additional file 3). No strong enrichment for functional categories was evident, apart from an overlap with genes that are strongly periodically regulated during the cell cycle (P ~ 4 × 10^-7^) [37,38]. Most of the 16 overlapping genes function in mitosis or cell division and could be important for stress recovery to re-start the cell proliferation after H_2_O_2_-induced arrest.

The 123 genes of cluster 2 showed the reverse trend to cluster 1 genes: they were translationally down-regulated, while their mRNA levels were up-regulated (Figure 2a; Additional file 3). Cluster 2 genes were enriched for the Gene Ontology (GO) [39] terms 'oxidoreductase activity' (P < 1 × 10^-8^) and 'amino acid biosynthesis' (P ~ 3 × 10^-10^). Cluster 2 was also enriched for genes highly expressed at transcriptional and translational levels in unstressed cells [36,40]: they showed higher mRNA levels (P < 1 × 10^-8^), higher RNA polymerase II occupancy (P ~ 8 × 10^-3^), longer mRNA half-life and polyA tails (P ~ 7 × 10^-3 ^and 2 × 10^-4^, respectively) as well as higher ribosome occupancy and density (P < 1 × 10^-8 ^and 4 × 10^-3^, respectively) compared to all mRNAs in unstressed cells. Shorter mRNAs are more efficiently translated in unstressed cells [13], but cluster 2 genes were not biased with respect to mRNA size. It is possible that the antagonistic translational down-regulation, which is maximal at 60 minutes after stress induction, balances protein production of these highly expressed genes for eventual stress recovery.

The 208 genes of cluster 3 showed a transient boost in translation in response to heat, but their mRNA levels were all down-regulated (Figure 2b; Additional file 3). Cluster 3 was strongly enriched for genes encoding ribosomal proteins (P ~ 3 × 10^-147^). In contrast, in response to oxidative stress, genes for ribosomal proteins showed similar translation profiles to unstressed cells at 15 minutes before becoming strongly down-regulated at the translational level at 60 minutes. Cell growth and proliferation are tightly linked to ribosome biogenesis [41]; the translational up-regulation of ribosomal protein genes at 15 minutes in heat stress could therefore reflect a transient boost in growth in response to the shift from 32°C to 39°C, as it takes some time to reach temperature equilibrium and *Schizosaccharomyces pombe *shows the fastest growth at approximately 35°C. At 60 minutes in heat stress, however, most of the cluster 3 genes became translationally down-regulated (Figure 2b), probably reflecting subsequent stalling of growth at 39°C. Intriguingly, a minority of seven ribosomal protein genes did not become transiently induced at the translational level in heat (*rpl301*, *rpl302*, *rpl401*, *rpl402*, *rpl501*, *rps401*, *rps403*), which might reflect functional specialization of different ribosomal proteins as suggested for budding yeast [42].

### Concordant changes at mRNA and protein levels for induced but not for repressed genes

**Figure 4:**
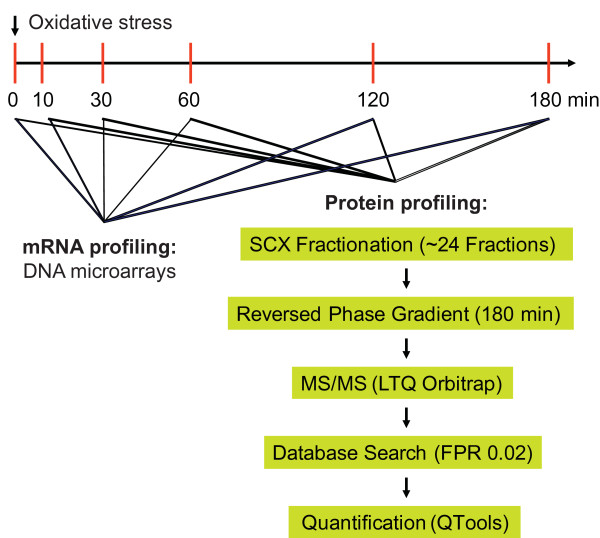
**Proteome profiling during oxidative stress**. Scheme delineating the experimental procedures applied to measure protein levels at different time points immediately before and after exposure to H_2_O_2_. Cells were harvested at the indicated time points, followed by preparation of protein lysates and digestion of proteins into peptides. Peptides were separated by strong ion exchange SCX chromatography into 24 fractions. Each fraction was separated by reversed phase chromatography and directly eluted into a ThermoFisher LTQ Orbitrap mass spectrometer (for details see Materials and methods). Transcript levels were determined from the same samples using DNA microarrays. Proteins and mRNAs were measured in two independent biological repeats of the time-course experiment, and quantified using QTools [60]. MS/MS, tandem mass spectrometry.

Given that changes in mRNA levels and in translation were largely coordinated, we expected that changes in protein levels mostly reflect changes in mRNA levels. We applied a proteomics approach to determine the relative changes in protein levels at multiple times after addition of oxidative stress compared to unstressed cells (Figure 4). The same samples were also interrogated with microarrays for mRNA expression profiling. Two independent biological repeats were performed for protein and mRNA profiling. We could obtain spectrum count data for 4,644 *S. pombe *proteins in at least one sample, and could detect 2,147 proteins in all 12 samples of both repeats (minimum of two identified unique peptides per protein; Additional file 4). Of these 2,147 proteins, 234 (11%) showed significant changes in abundance during the stress time course (Materials and methods).

**Figure 5:**
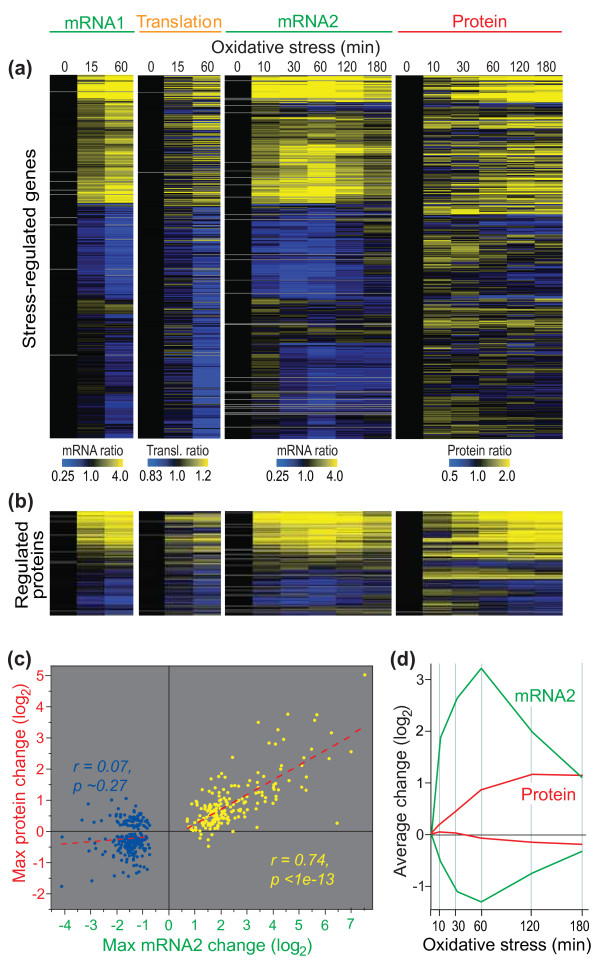
**mRNA, translation, and protein regulation during oxidative stress**. **(a) **Hierarchical cluster analysis with columns representing experimental time points and rows representing 811 genes whose mRNAs showed significant expression changes after exposure to H_2_O_2 _and whose proteins could be detected in all conditions. mRNA1: mRNA expression relative to the unstressed samples is color coded as indicated at the bottom, using same samples as for the translation experiment (Figure 2). Translation: translational efficiency relative to the unstressed samples is color coded as indicated at the bottom. mRNA2: mRNA expression relative to the unstressed samples is color coded as indicated at the bottom, using the same samples as for the proteome experiment. Protein: protein expression relative to the unstressed samples is color coded as indicated at the bottom. Average data of biological repeats are shown, with missing data in gray. **(b) **Cluster analysis as in (a) for the 232 genes that encode proteins showing significant changes in expression in the proteome experiment and with data in >50% of all conditions used for clustering. **(c) **Scatter plot showing linear regressions of the maximum average log_2 _changes in mRNA and protein expression across the time-course experiment mRNA2/Protein shown in (a) for proteins that were detected in all conditions. Yellow dots, 193 genes showing >1.5-fold induction in mRNA expression after exposure to H_2_O_2 _in at least 4 of 7 stress time points in experiments mRNA1 and mRNA2 shown in (a); blue dots, 226 genes showing >1.5-fold repression in mRNA expression after exposure to H_2_O_2 _in at least 4 of 7 stress time points in experiments mRNA1 and mRNA2. Pearson's correlations and probabilities (two-tailed test) are indicated for induced (yellow) and repressed (blue) mRNAs. **(d) **Graph showing average mRNA (green) and protein (red) expression profiles (log_2 _ratios) in the experiment mRNA2/Protein shown in (a). Solid and dashed lines indicate average profiles for 193 and 226 genes showing >1.5-fold induction or repression, respectively, in mRNA expression after exposure to H_2_O_2 _in at least 4 of 7 stress timepoints in experiments mRNA1 and mRNA2 shown in (a).

Figure 5a shows the expression profiles of all genes whose mRNA abundance was regulated during oxidative stress and whose proteins could be detected in all 12 samples. The data for transcriptome profiling in the two experiments (mRNA1 and mRNA2, performed with different microarray platforms and in different laboratories) were highly similar overall. Moreover, changes in mRNA and translation profiles were largely mirrored by changes in protein profiles, especially for up-regulated transcripts (Figure 5a). The inverse analysis, starting from proteins whose profiles significantly changed during oxidative stress, showed a similar picture of highly concordant up-regulation at multiple regulatory levels (Figure 5b). While the genes up-regulated at the mRNA level produced up-regulation of the corresponding proteins, the down-regulated mRNAs showed much weaker relationships with protein profiles (Figure 5a,b). Accordingly, the linear correlation between maximal mRNA and protein changes was highly significant for up-regulated mRNAs, while there was no correlation for down-regulated mRNAs (Figure 5c). This pattern was also evident from the average mRNA and protein expression profiles (Figure 5d).

Although we limited our analysis to those proteins that were detectable in all conditions, the poor correlation between down-regulated mRNAs and corresponding proteins could reflect that low abundance proteins are less reliably quantified by mass spectrometry. On the other hand, it is plausible that this poor correlation reflects the biological reality of protein regulation, as proteins with long half lives are expected to maintain stable expression for some time after shutting down production. Notably, similar findings were recently reported by Lee *et al*. [33] during osmotic stress in budding yeast. These authors applied mathematical modeling to their transcriptome and proteome data sets, suggesting that reduction in transcript abundance may serve to redirect ribosomes to newly produced mRNAs.

The dynamic range for regulation of mRNA abundance was substantially larger than for regulation of protein abundance (Figure 5a,b,d). Similar results were obtained in a recent study in budding yeast applying a different proteomics approach [33]. Moreover, while most up-regulated mRNAs transiently peaked in expression at 60 minutes after stress induction and then decreased again, the corresponding proteins showed a delayed and gradual increase in expression up to 180 minutes (Figure 5a,b,d). The delayed up-regulation of proteins may reflect the time required for translation along with protein half-lives. Similar mRNA and protein expression patterns have recently been observed in budding yeast [33,43]. Lee *et al*. [33] have shown that the 'burst' in mRNA expression serves to accelerate protein expression before mRNA levels adjust to maintain a new steady-state.

**Figure 6:**
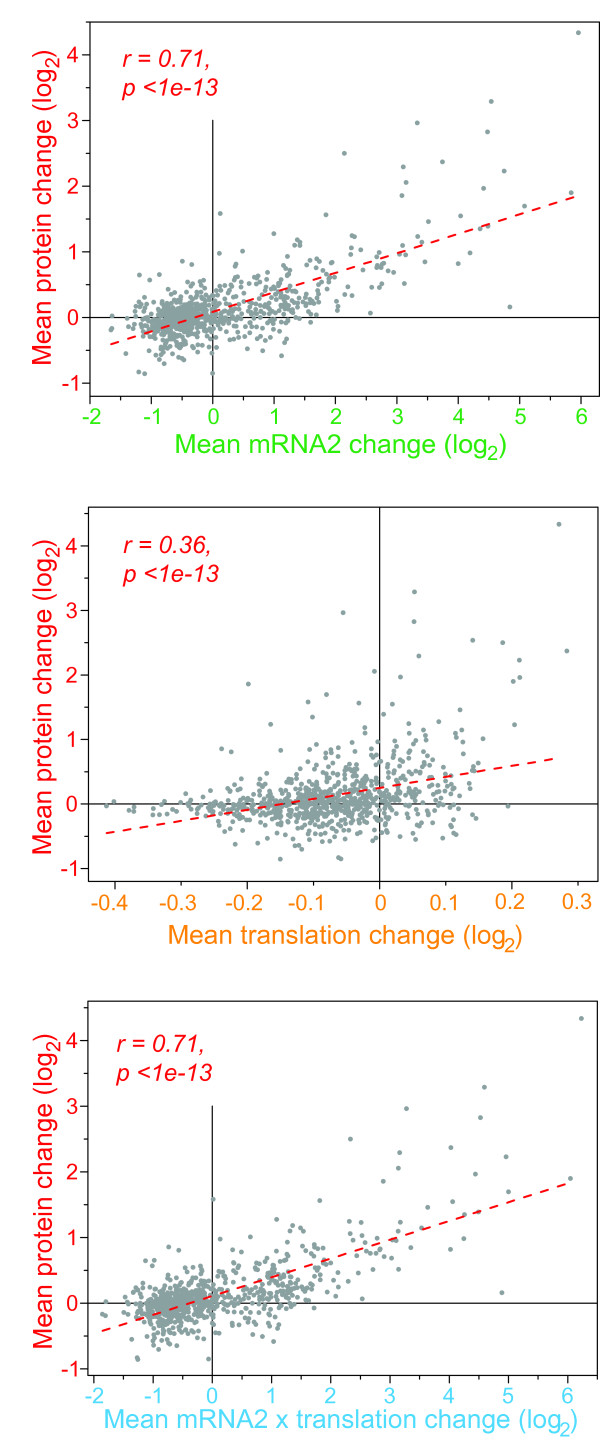
**Relationships between mRNA, translation, and protein regulation**. Scatter plots showing linear regressions of the average log_2 _changes of proteins and of average changes in mRNA2 experiment (top; Figure 5), average changes in translation (middle), and combined changes in mRNA2 and translation (bottom; product of average changes). The gene list is the same as used in Figure 5a. The Pearson's correlations and probabilities (two-tailed test) are indicated for each comparison.

Overall, the average changes in protein expression were substantially correlated with corresponding changes in mRNA expression and, to a lesser extent, with changes in translation (Figure 6). Factoring in both transcription and translation by simply multiplying relative mRNA and translational changes, however, did not further improve the correlation with protein expression (Figure 6). The global correlation between mRNA and protein expression we observed here is stronger than the more modest correlations reported in previous papers [24-32], but is comparable to higher correlations reported in some smaller-scale studies [44,45] and recent global studies [33,34].

To explore any effects of post-transcriptional regulation during oxidative stress, we identified genes that went against the overall trend of concordant regulation at mRNA and protein levels. Only 22 and 19 proteins showed increased and decreased expression, respectively, in the absence of changes in mRNA expression (Additional file 5). Translation of the corresponding mRNAs was not strongly regulated, however. These data raise the possibility of additional regulation at the protein level, by stress-induced protein stabilization or degradation. A higher number of proteins did not show significant expression changes, although the corresponding mRNAs showed increased or decreased expression (63 and 156 mRNAs, respectively; Additional file 5). It is likely that the latter reflect the overall limited correlation between down-regulated mRNAs and protein expression discussed above (Figure 5). However, some of these discrepancies could also be explained by compensatory regulation at the level of translation, especially for the mRNAs with increased expression: 16 of the 63 up-regulated mRNAs were translationally down-regulated (cluster 2 genes in Figure 2a), but only 2 of the 156 down-regulated mRNAs were translationally up-regulated (cluster 1 genes in Figure 2a). These patterns suggest that, in some cases, translation is regulated to counter the stress-induced changes in mRNA expression so that the resulting protein expression does not substantially change. It is possible that the proteins encoded by these mRNAs are not immediately required under the given condition but are prepared at the mRNA level to become rapidly available on short notice ('translation on demand') [46]. The majority of cluster 1 and 2 genes showed changes in protein expression consistent with the changes in mRNA expression, indicating that, in most cases, transcriptional regulation dominates translational regulation. This conclusion is supported by the findings that mRNA expression correlated better than translational changes with protein expression, and factoring in translation did not improve the overall correlation (Figure 6). Finally, a few proteins showed abundance changes in the opposite direction to their mRNAs: 20 proteins increased and 12 proteins decreased in expression, while the corresponding mRNAs decreased and increased, respectively (Additional file 5). While these few exceptions could reflect technical noise, it was striking that several ribosomal proteins and translation factors showed such opposite regulation. This finding raises the possibility of some remodeling of the translation machinery via regulation of protein stability and the involvement of specialized ribosome subunits during stress [42].

## Conclusions

This study analyzed the global changes in mRNA, translation, and protein profiles in response to environmental stress in fission yeast. We observed strong overall concordance between changes in mRNA and changes in translation, for both induced and repressed genes, in response to three different insults: oxidative stress, heat shock, and DNA damage caused by MMS. We have previously reported that mRNAs that are efficiently transcribed are also more efficiently translated under an unstressed steady-state condition, leading to a 'rich getting richer' effect [36]. The data presented here indicate that transcriptional changes during stress are often accompanied by translational changes in the same direction. Such homo-directional changes in transcription and translation (termed 'potentiation') have also been reported in budding yeast [18,47,48]. How might such coordination of transcription and translation be achieved? The RNA polymerase II subunits Rpb4 and Rpb7 shuttle from the nucleus to the cytoplasm where they promote efficient initiation of translation [49], which provides a potential mechanism to integrate transcriptional and translational regulation. Moreover, in fission yeast, the translation factor Int6 and the MAPK Sty1 are involved in both transcriptional and translational responses to stress [50-54], although the detailed mechanisms are not clear. Finally, there is strong evidence that translational repression and mRNA decay are linked [55,56], which could also contribute to the coordinated regulation of mRNA expression and translation.

Besides the overall concordant regulation of transcription and translation, we identified gene clusters that were antagonistically regulated at the levels of mRNA and translation. Around 200 genes each showed such antagonistic regulation in oxidative and heat stress, but the types of genes and the patterns of regulation were unique for each stress. These data highlight the sophistication and fine-tuning of stress-regulated gene expression, which may promote a balanced and coordinated protein production for stress survival and recovery.

We also determined relative changes in protein abundance during oxidative stress, with approximately 43% of all fission yeast proteins producing robust data. Changes in protein expression were delayed compared to changes in mRNA expression, but we observed a strong overall concordance between mRNA and protein profiles, but less so between translation and protein profiles. Moreover, the correlation between mRNA and protein expression did not significantly improve after factoring in translation, and protein expression tended to reflect mRNA expression in cases where transcription and translation were regulated antagonistically. We therefore conclude that transcription dominates gene regulation during oxidative stress, and most variance in protein expression can be explained by changes in mRNA expression. Under the condition analyzed, post-transcriptional control may therefore be mainly applied to support transcriptional control, leading to a more robust and coordinated response, but also to regulate groups of specialized genes.

Notably, only up-regulated mRNAs showed a strong correlation with protein expression, while down-regulated mRNAs showed no such correlation. Similar findings were recently reported for osmotic stress in budding yeast [33]. These authors propose that reduction in transcript abundance may serve to redirect translational capacity to newly produced mRNAs rather than to reduce protein levels. This intriguing concept could also apply to fission yeast given the large number of transcriptionally and translationally up-regulated mRNAs during oxidative stress, which may lead to limitation of ribosomes and/or translation factors in the absence of any balanced down-regulation of mRNAs. In fact, the concordant mRNA and translation profiles reported here indicate that ribosomes are actively re-distributed from lowly to highly expressed transcripts. For long-lived proteins, the cells can transiently down-regulate the corresponding mRNAs without greatly affecting protein abundance, especially in stressed cells that show growth arrest. The findings that reduced mRNA expression does not necessarily lead to reduced protein expression during stress in two evolutionarily distant yeasts has important implications for transcriptome analyses, as reduced mRNA expression is typically interpreted to reflect reduced expression, and dispensability, of the corresponding proteins.

## Materials and methods

### *S. pombe *strains and growth conditions

All stress experiments were performed with wild-type strains (972 *h^- ^*for the translational profiling experiments, DS448/2 *h^- ^leu1-32 ura4-d18 *for the proteomics experiments). Unless indicated otherwise, cells were exponentially grown at 32°C in rich medium (supplemented yeast extract medium [57]). Oxidative stress was induced by the addition of H_2_O_2 _(Sigma-Aldrich, Gillingham, Dorset, UK) to a final concentration of 0.5 mM, DNA damage was induced by the addition of MMS (Sigma-Aldrich) to a final concentration of 0.02 % v/v, and heat stress was induced by moving the culture flask with growing cells from 32°C to a 39°C water bath.

### Translational profiling

To study translational regulation in response to environmental stress, medium-resolution polysome profiling was performed. Preparation of cell lysates and polysome fractionation was done as described [36], with the modification that cycloheximide was added directly to a final concentration of 100 μg/ml when cells were harvested. Twelve fractions were collected during polysome fractionation. Fractions 1 to 3, 4 to 6, 7 to 9, and 10 to 12 were combined, respectively, into four pools. RNA from each pool was precipitated overnight at -20°C after the addition of an equal volume of 100% ethanol. After centrifugation at 4°C for 90 minutes, the pellets were air dried and dissolved in 100 μl DEPC-treated water. The RNA was then purified using RNeasy columns (Qiagen, Crawley, West Sussex, UK), eluted with 30 μl DEPC-treated water, and 10 μl of RNA from each of the four pools was labeled using a mix of oligo(dT)-primers and random hexamers, the SuperScript™ Direct cDNA Labeling System (Invitrogen, Grand Island, NY, USA), and Cy3/Cy5-dCTP (GE Healthcare, Chalfont St Giles, Bucks, UK). The labeled RNA from each pool was hybridized against labeled genomic DNA as a reference onto *S. pombe *spotted microarrays containing all known and predicted genes and normalized using a customized script as described [58]. DNA labeling was performed using the Bioprime^® ^DNA labeling system (Invitrogen) and Cy3/Cy5-dCTP (Amersham) according to the manufacturer's instructions. Labeled DNA was purified using the QIAquick PCR purification kit (Qiagen) or the Illustra CyScribe GFX purification kit (GE Healthcare) and was eluted in a volume of 100 μl. One separate control was used for each round of translational profiling. The numbers of independent biological repeats were as follows: untreated control, 4 repeats; 15 minutes H_2_O_2_, 3 repeats; 60 minutes H_2_O_2_, 1 repeat; 15 minutes MMS, 2 repeats; 60 minutes MMS, 1 repeat; 15 minutes 39°C, 2 repeats; 60 minutes 39°C, 1 repeat.

### Analysis of translational profiling data

Microarray data from each of the four pools were processed and normalized using a customized normalization script [58]. For each mRNA with data in all four pools, translational profiles were calculated as the percentages of a given mRNA in each of the four pools such that the total overall pool was 100%. Two automated approaches were used to initially identify altered translational profiles to compare stress conditions with the corresponding control (Figure 1c). The two methods have complementary advantages and were used to cast a wide net of potential genes that are translationally regulated, which were subsequently visually inspected to generate conservative 'curated' lists of translationally regulated genes. First, the total difference between the two corresponding profiles was calculated by determining the sum of the absolute values of the differences for each of the four data points for a given mRNA, comparing the stress and the control profile and adding up the four values. A small total difference reflects similar translational efficiency and profiles in stressed and unstressed cells, whereas a high total difference reflects a change in translational efficiency and profile. Second, a translation ratio for each mRNA in each condition was calculated by multiplying the percentages in each fraction with arbitrary relative factors of 0.1, 0.2, 0.3 and 0.4 for fractions 1 to 4, respectively, to give higher weight for mRNAs in the fractions with more ribosomes. The results for each mRNA were then summed to obtain scores ranging from 10 to 40. The translation ratio was then obtained by dividing the score of a given mRNA in a stress condition by the score of the same mRNA in the control condition. A translation ratio >1 reflects a translational up-regulation, whereas a translation ratio <1 reflects a translational down-regulation. The translation ratio was independently calculated for each stress sample and the corresponding control sample, and used to visualize translational regulation in the cluster analysis of Figure 2. All data for percentage RNA in each fraction, sum of differences, scores and translation ratios are provided in Additional file 1. The sum of differences and the translation ratios were then separately averaged for samples of which we had independent repeats. After exploring different thresholds, cutoffs of 30 (sum of differences between profiles) and 1.15 (translation ratio) were applied, and genes that passed the cutoffs from either criteria were pooled to generate a combined set of genes. Translational profiles from this combined gene set were then visually inspected to generate a conservative, curated data set of translationally regulated mRNAs. The criteria used for visual inspection were as follows. The polysome profiles had to be similar among different repeats of control and stress samples, but had to be consistently different between the control and the stress samples. Moreover, a down-regulation in translation had to be reflected by both an increase of a given mRNA in fractions 1 to 2 and by a concomitant decrease in fractions 3 to 4, whereas an up-regulation in translation had to exhibit the inverse trend.

### mRNA expression profiling

To measure changes in mRNA expression for the samples used for translational profiling, labeled total mRNA from stressed cells was competitively hybridized against labeled total mRNA from unstressed control cells on the same spotted microarrays [58]. RNA extraction, microarray hybridization, washing, and scanning was done as described [36,58]. RNA labeling was performed using the SuperScript™ Direct cDNA Labeling System (Invitrogen) with a mix of oligo(dT)-primers and random hexamers, and Cy3/Cy5-dCTP (Amersham) according to the manufacturer's instructions, except that 2 μl instead of 3 μl of Cy-dyes per labeling reaction were used. Processing and normalization of microarray data were done using our standard script [58]. In the case of one time course in response to H_2_O_2_, labeled total mRNA from each time point (0, 15, and 60 minutes) was competitively hybridized to a pooled reference sample, consisting of a mixture of mRNA from all time points, and microarray data were then normalized to time point 0 (control).

To measure changes in mRNA expression for the samples used for proteomics, cells were harvested by centrifugation, cells were washed in a buffer containing 150 mM NaCl, 10 mM EDTA, 50 mM NaF and 1 mM NaN3, and subjected to RNA isolation as follows: 5 ml RNAzol (Tel-Test, Inc., Friendswood, Texas, USA) preheated to 65°C and 1 ml silica beads (BioSpec, Bartlesville, Oklahoma, USA) were added to the cell pellets, and the tubes were subjected to three cycles of 2 minutes vortexing, followed by 5 minutes heating at 65°C. Chloroform (500 μl) was added, followed by 5 minutes incubation on ice and centrifugation for 30 minutes at 5,000g. Then, 2.5 ml of the aqueous layer was removed, followed by addition of 2 ml isopropanol and incubation on ice for 20 minutes. The samples were spun at 5,000g for 30 minutes, and the liquid was removed by vacuum aspiration. The resulting pellets were washed with cold 80% ethanol, dried and resuspended in RNAse-free water. Total RNA (100 μg) of each sample was further purified using an RNAeasy kit (Qiagen) following the manufacturer's protocol. RNA was reverse transcribed with Superscript II RT (Invitrogen), in the presence of aminoallyl-dUTP (dUTP:dTTP = 3:2) in reactions primed with oligo-dT. Following cleanup via QIAquick column (Qiagen) in Tris-free buffers, cDNAs were coupled to NHS-CyDye (GE Healthcare) in bicarbonate buffer. Coupled cDNAs were purified to remove uncoupled dye via QIAquick column purification. Cy3-labeled total RNA samples were competitively hybridized on cDNA microarrays [59] with Cy5 labeled RNA pooled from all six time points. A biological replicate was performed and analyzed as a dye swap (pool = Cy3, time series = Cy5). Datasets were normalized and log_2 _ratios were determined relative to the 0 minute time point.

### Statistical analyses

The clustering analyses in Figure 2 were performed using Gene Tree Clustering based on average linkage in GeneSpring GX (Agilent, Santa Clara, California, USA) with the Spearman correlation as similarity measure. Enrichments for functional lists or Gene Ontology terms [39] were determined using the hypergeometric distribution. Spearman rank correlations (r) and corresponding *P*-values were calculated using the cor.test function in the statistics package R (version 2.2.1). Pearson's correlations and probabilities (two-tailed test) were calculated in OriginPro 8.5 (OriginLab Corporation, Northampton, Massachusetts, USA).

### Two-dimensional liquid chromatography-tandem mass spectrometry analysis and protein quantification

Duplicate cell cultures were exposed to 0.5 mM H_2_O_2 _for the times indicated in Figure 4. Preparation of cell lysate, trypsin digestion, two-dimensional liquid chromatography-tandem mass spectrometry analysis, and database searching were performed exactly as described previously [60]. In total, 4,644 *S. pombe *proteins were identified based on a minimum of 2 unique peptides in at least 1 of the 12 samples. A subset of 2,147 proteins, which were identified in each of the 12 samples of the time course, were used for quantitative comparisons. First, a control was generated by averaging the spectrum count of each time point. The data for each time point were compared with the control in a linear least squares fit statistic, which was then used to calibrate each time point. After this normalization step, a dataset was generated by computing the log_2 _ratios of the expression data and the untreated reference (0 minutes). A quadratic-linear regression method was used to identify proteins with significantly changed temporal expression patterns (determined by F-statistics and least squares estimates) [61]. A major advantage of this approach is that it not only preserves the order of the time points but also identifies differentially expressed proteins and classifies these proteins based on their temporal expression profiles. The 234 significantly changed proteins (*P *< 0.05) were classified into four categories based on the expression profiles using an Excel macro (linear up, linear down, quadratic convex, quadratic concave) [61]. Duplicate values for each time point were averaged to obtain a single value for each protein at each time point. All data are contained in Additional file 3.

### Accession numbers

All microarray data have been submitted to ArrayExpress under accession numbers [E-MTAB-851] (translational profiling experiments) and [E-MTAB-891] (proteomics experiments).

## Abbreviations

CESR: core environmental stress response; MAPK: mitogen-activated protein kinase; MMS: methylmethane sulfonate.

## Competing interests

The authors declare that they have no competing interests.

## Authors' contributions

DHL, MWS, and SW carried out the genomic and proteomic experiments, and participated in the study design and in data analysis. DAW and JB conceived the study, and participated in the study design and in data analysis. JB drafted the manuscript. All authors read, revised and approved the final manuscript.

## Supplementary Material

Additional file 1 **Data of all mRNA and translation ratios (Figure **2**), and data from translational profiling analysis (percentage RNA in each fraction, sum of differences, and scores (Figure **1c**)**. Click here for file

Additional file 2 **Annotated list of genes in clusters 1 to 3 (Figure **2**)**. Click here for file

Additional file 3 **Annotated list of translationally regulated genes in three stress conditions (Table **1**)**. Click here for file

Additional file 4 **Proteomics data including raw spectrum count measurements, normalized averaged data and list of significantly changed proteins; mRNA data obtained in parallel are also included**. Click here for file

Additional file 5 **Annotated list of genes showing discordant regulation at mRNA and protein levels**. Click here for file
